# A new future for ecancer

**DOI:** 10.3332/ecancer.2009.ed4

**Published:** 2009-05-15

**Authors:** Linda Cairns

**Affiliations:** Scientific Editor, *e*cancermedicalscience, European Institute of Oncology, Milan, Italy

I am delighted to announce that *e*cancer has been selected by the National Library of Medicine (NLM) to be included in PubMed Central and indexed in PubMed, the world's most heavily used set of databases for health and medical professions.

All articles will be indexed in PubMed and will remain as open access material online in *e*cancer. Joining PubMed, after such a short history, emphasises the quality of the journal.

In close collaboration with the journals managing editor Prof Gordon McVie we have endeavored to reach as many people as possible involved in cancer care and research in an effort to enhance cancer education around the globe.

These efforts have been made possible by the unwavering support of Prof Umberto Veronesi, who has for many years wished to establish a completely open access cancer journal which is free to doctors and researchers everywhere.

Obtaining indexing with PubMed was a tremendous undertaking and the future now looks bright for *e*cancer. We will continue our mission of bringing cancer information to a global audience and aim to make *e*cancer the logical place to disseminate all oncology research and information.

The next phase in the life of *e*cancer brings new responsibilities. The journal must continue to publish high quality papers and I am extremely proud of the excellent standard. They are a principal reason why we gained entry into PubMed. However innovative research from Institutes around the world often still goes unpublished. I encourage you to share even negative results with your colleagues and submit your experiences to *e*cancer.

I must also thank our editorial board members, authors, and peer-reviewers. *e*cancermedicalscience was established and is currently running thanks to the financial support of Umberto Veronesi Foundation, European CanCer Organisation (ECCO), The European Institute of Oncology (IEO) and the IEO Foundation.

*e*cancer is also available in the online databases SCOPUS and EMBase.

We look forward to publishing more papers, interviewing more researchers for ecancertv, and continuing to contribute to the progress against cancer,

